# Patient house calls in Attica and Thessaloniki, Greece (2005-2015): a model for out-of-hospital multispecialty emergency medicine

**DOI:** 10.1186/s12913-018-3111-0

**Published:** 2018-04-27

**Authors:** George Theocharis, Spyridon G. Barbas, Theodore Spiropoulos, Petroula E. Stamouli, Dimitrios N. Perdikis, Matthew E. Falagas

**Affiliations:** 1 SOS doctors, Athens, Greece; 2 Department of Medicine, “Euroclinic” General Clinic, Athens, Greece; 3 Alfa Institute of Biomedical Sciences (AIBS), 9 Neapoleos Street, 151 23 Marousi, Greece; 4 Department of Applied Mathematical and Physical Sciences, National Technical University of Athens, Athens, Greece; 5 Department of Medicine, Henry Dunant Hospital Center, Athens, Greece; 6 Department of Medicine, Tufts University School of Medicine, Boston, Massachusetts USA

**Keywords:** Home visit, Frailty, Elderly, Disability

## Abstract

**Background:**

The SOS-doctors are a network of physicians who perform house-call visits in the areas of Attica and Thessaloniki, Greece.

**Methods:**

Patients requesting medical services by the SOS doctors during the period 1/1/2005 – 31/12/2015 were eligible for inclusion in this retrospective analysis.

**Results:**

During this period 335, 212 home visits were performed. Females used this service more frequently compared to males (60.5% versus 39.5%). Among the age-groups, patients aged over 75 years made 56.6% of all house calls. Fewer phone requests were recorded during autumn than in winter (21.1% versus 29.1%). Infections were the most common cause of house-visits (29%), followed by cardiovascular diseases (10.3%), musculoskeletal (9.1%), gastrointestinal (6.3%) and neurological disorders (3.7%). An increasing demand for radiology at home was observed, starting at 352 calls in 2009 and reaching 2230 in 2015. Finally, 9.2% of patients were advised to be admitted into a hospital.

**Conclusion:**

A shift towards older age, but not the oldest old (> 90 years), and acute conditions was observed during the study period. The study confirms that home visits retain a significant role in the modern health care systems.

## Background

Home care has been traditionally considered an important mode of health care service. Although differences in practice among geographical areas and health systems have been described [1], a continuous decline in home visits was observed throughout the 20th century [2]. Changes in the availability of transportation means, technology-assisted diagnosis and monitoring, physicians specialization, and payment systems resulted in practice alterations [1]. The interest was renowned in the 21st century and will probably increase further due to disability and frailty of the aging population, increased pressure on health care economics, as well as advances in healthcare technology that created unprecedented opportunities to combine the delivery of high quality healthcare services and the comfort of patients’ homes.

Modern home visits require the availability of both physicians and paramedical staff. Four main modalities for house calls can be identified; routine primary care visits including prevention programs, calls for acute illnesses or exacerbations of chronic conditions, post-hospitalization intravenous/parenteral treatment, and hospice and palliative care. When accomplished, home visits are expected to improve quality of care and increase patient satisfaction [3, 4], achieve optimal health outcomes for patients [5, 6], reduce medication errors and falls [7, 8], avoid unnecessary hospitalizations or re-admissions [9, 10], and ultimately reduce healthcare cost [11]. A home visit can enhance the relationship between a physician and the patient, and enable the physician’s understanding of the patient’s environment and support systems [2].

In previous analyses of SOS doctors’ data we described the characteristics of patients requesting home visits as well as physician practice [12, 13]. We also described the utilization of parenteral antibiotic treatment at home in the acutely ill patients as well as their outcomes [14]. In the current analysis we sought to update this data and provide further insights on the demand of physician home visits in Greece. We studied trends in utilization of the service according to patient characteristics, time (within the day and the entire year), trends in the requested specialty, etiology of the house-call and patient satisfaction.

## Methods

Patients requesting medical services by the SOS doctors in the areas of Attica and Thessaloniki, Greece, during the period 1/1/2005 – 31/12/2015 were eligible for inclusion in this retrospective analysis. The SOS doctors are a network of physicians of most clinical specialties (internal medicine, cardiology, pneumonology, neurology, hematology, dermatology, gastroenterology, pediatrics, psychiatry, ophthalmology, otorhinolaryngology, anesthesiology, surgery, orthopedics, urology, gynecology, dentistry, stomatology, microbiology, radiology) who perform house-call visits. The service is provided in the greater metropolitan areas of Athens (with a population of approximately 4.2 million citizens) and Thessaloniki (with a population of approximately 0.8 million citizens); other areas of the prefectures of Attica and Thessaloniki are also covered to a lesser extent. A physician answers to phone calls of patients requesting medical services and makes the initial assessment whether the patient should be seen by a doctor and determines the most appropriate specialty for the case. The service is available 24 hours a day, 365 days a year. If the house-call visit is carried out, the doctor fills a specially designed form with data regarding current illness, past medical and surgical history, findings of the physical examination, assessment based on the history and examination, likely diagnosis, and management plan that includes medical and/or surgical treatment, laboratory and/or imaging tests, re-evaluation and/or commendation for hospital admission. A follow up phone call is randomly performed by a secretary up to 15 days after the specialist’s visit to record patient satisfaction regarding the physician’s performance. The secretary used a pre-specified 4 item questionnaire for satisfaction in 4 main domains: time to arrival, communication, diagnostic accuracy and management.

Most physicians arrive to the patient’s home using their own vehicle within approximately 30-60 min. All physicians are required to carry medical supplies and diagnostic tests or devices according to their specialty. In addition, 3 cars equipped with diagnostic and therapeutic devices (point of care diagnostic blood tests, ultrasound, x-rays, electrocardiograph, defibrillator) are also available 24 h a day, 365 days a year. When needed, a network of specialized homecare nurses is available to support the physicians’ home-visit and administer treatment. All expenses (including physician’s visit, diagnostic tests and treatment) are covered by the patient or occasionally by their private insurance plan.

The Ethics Committee of SOS doctors, Greece approved the study for being quality improvement. Due to the retrospective and observational nature of the study written informed consent was not required. The available data regarding home visits by SOS doctors during the study period were stratified based on patients’ demographics (age and sex) and time of the visit (time within the day, month, season, and year) to recognize patterns of utilization of this health-care service. In addition, the frequency of diseases for which home-visits were performed as well as the recommendations of the SOS doctors regarding the need for hospitalization were analyzed. The association between the distribution of calls to the SOS network and the distribution of the Attica population according to pre-specified age groups was evaluated using correlation coefficient. Analyses were performed using SPSS software program (version 17.0, SPSS Inc., Chicago, Illinois, USA). A *p* value < 0.05 was set to denote statistical significance for all analyses.

## Results

During an 11-year period SOS doctors performed 335,212 home visits, upon phone requests. Table 1 presents data regarding age, gender, and time related to the call. The majority of these calls (99.5% of house-calls) were received from the area of Attica. Females used this service more frequently compared to males (60.5% versus 39.5%).

**Table 1:** Characteristics of house-call visits in Attica and Thessaloniki, Greece (observation time period 2005-2015)

Characteristic	N of house-calls	% of all house-calls
Gender		
Female	202,859	60,5
Male	132,353	39,5
Age-Group		
1-15	9324	2,8
16-30	13,703	4,1
31-45	33,035	9,9
46-60	37,449	11,2
61-75	45,705	13,6
76-90	115,344	34,4
> 90	74,350	22,2
non-recorded	6302	1,9
8-Hour		
00-08	41,832	12,5
08-16	156,961	46,8
16-00	136,419	40,7
Month		
January	34,767	10,4
February	31,531	9,4
March	33,624	10,0
April	29,505	8,8
May	26,826	8,0
June	24,710	7,4
July	24,554	7,3
August	27,724	8,3
September	21,285	6,3
October	24,342	7,3
November	24,947	7,4
December	31,396	9,4
Season		
Winter	97,694	29,1
Spring	89,955	26,8
Summer	76,988	23,0
Fall	70,574	21,1
Year		
2005	20,056	6,0
2006	22,733	6,8
2007	29,546	8,8
2008	33,047	9,9
2009	37,052	11,1
2010	34,208	10,2
2011	35,774	10,7
2012	34,288	10,2
2013	29,865	8,9
2014	29,191	8,7
2015	29,452	8,8

**Fig. 1:**
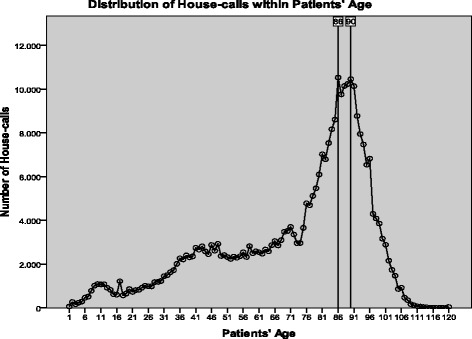
Distribution of calls within age

**Fig. 2:**
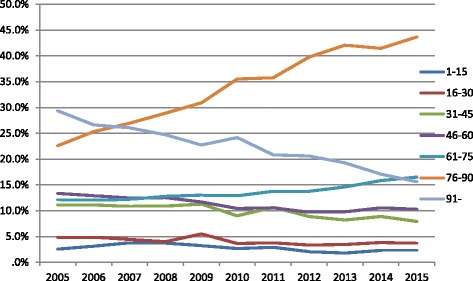
Proportion of home-visits according to age groups during the study period

**Fig. 3:**
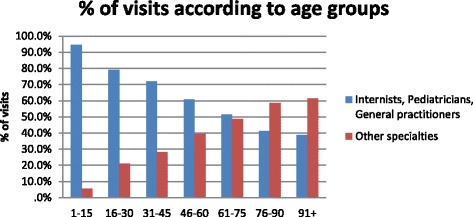
The distribution of home visits according to the physician’s specialty in different age groups

Among the age-groups, patients aged over 75 made the 56.6% of all house calls. Specifically, patients between the age of 76-90 (34.4%) requested the most home-visits, followed by those aged > 90 years (22.2%). The highest number of calls was done by patients 86 and 90 years old (Fig. 1). On the contrary, those aged between 0 and 15 requested only 2.8% of the house-visits. The annual percentage of calls in the age group 76-90 years was increasing up to 2015, while the corresponding absolute number was increasing up to 2012 and then remained almost stable with minor ups and downs. In the age group > 90 years, the absolute number of calls up to 2009 was increasing, followed by a decrease thereafter. All other age groups showed a similar distribution in the absolute number of home-visits, besides the age group 61-75 that requested more visits annually. Moreover, it contributed for the first time more calls than the age group > 90 years in 2015 (Fig. 2). Regarding the requested specialty, the median age of patients examined by internists or general practitioners was 75 years, while that of the other specialties was 85 (*p* < 0.001). Figure 3 shows the distribution of home visits according to the physician’s specialty in different age groups. The number of home visits for internists and general practitioners declined with increasing patient age.

A correlation between the Attica population and the number of calls to the SOS network was observed for specific age groups (Table 2). Thus, there was a positive correlation for the age groups 15-29, 45-59, 60-74 and 75-89 and a negative correlation in the age groups 0-14 and > 90 years. In the age group > 90 a positive correlation in the period before the crisis and a negative after the crisis was observed. In addition, for the age group 74-89 the correlation was positive for the period before the crisis but no correlation was seen after the crisis (data not shown).

**Table 2:** Correlation between the percentage of calls and the percentage of population in each age group during the study period and according to the

Age group	*r*	*p* value	Correlation interpretation
> 90	−0.51	0.1	none
76-90	0.89	< 0.001	positive
61-75	0.98	< 0.001	positive
46-60	0.83	0.001	positive
31-45	0.21	0.54	none
16-30	0.72	0.01	positive
0-15	−0.83	0.002	negative

**Fig. 4:**
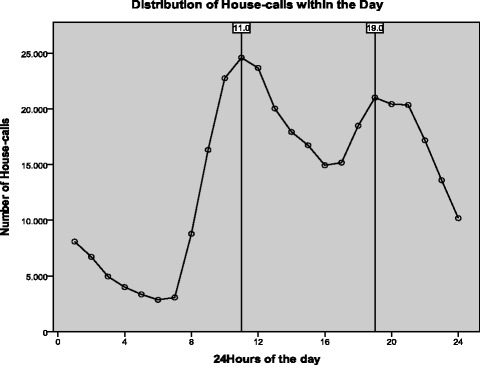
Distribution of calls within day

**Fig. 5:**
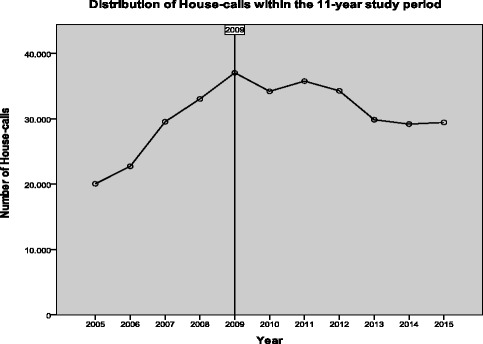
Distribution of calls within year

The distribution of house calls within the 24 h of the day demonstrates two peaks, one around 11:00 a.m. and a second around 7 p.m.; the fewest calls were recorded around 6:00 a.m. (Fig. 4). Regarding seasons, home-visits seem to be a periodic phenomenon which peak in winter months (29.1%) and decline during autumn months (21.1%). Only 6.3% of all calls were made during September, while the maximum proportion (10.4%) was recorded in January. The majority of house-calls (52.1%) were done between 2008 and 2012 (Fig. 5). The higher number of calls was recorded during 2009 (11.1%).

**Fig. 6:**
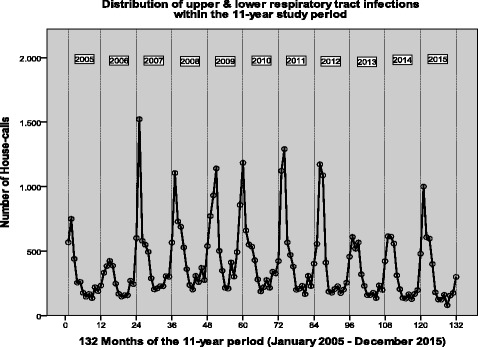
Distribution of upper and lower respiratory tract infections within the 11-year study period

Data regarding patients’ working diagnosis are presented in Table 3. The majority of calls were performed for acute conditions (80.7%); 11.7% were attributed to chronic conditions and 7.6% could not be classified as acute or chronic due to missing or inadequate data. Finally, 9.2% of patients were referred for hospital admission. Infections were the most common cause of house-visits (29%). Upper and lower respiratory tract infections comprise 19.4% of house-calls. In addition, respiratory tract infections seem to be the most significant reason of requesting a visit during winter (Fig. 6). Cardiovascular diseases comprised also a significant proportion (10.3%), followed by musculoskeletal (9.1%), gastrointestinal (6.3%) and neurological disorders (3.7%). It should be also mentioned that a significant proportion of home visits were done for symptoms that could not be linked to a specific working diagnosis as well as medical procedures like placement of urinary or nasogastric catheters.

**Table 3:** Working diagnoses for house-call visits in Athens and Thessaloniki, Greece (observation time period 2005-2015)

Diseases/Disorders	N of house-calls	% of all house-calls
Infections	97,293	29,02%
Upper respiratory tract infections	44,034	13,13%
Gastroenteritis	21,080	6,29%
Lower respiratory tract infections	19,320	5,76%
Urinary tract infections	7170	2,14%
Cardiovascular	34,665	10,34%
Hypertension	12,385	3,69%
Arrhythmias	4584	1,37%
Hypotension/Fainting/Syncope	4315	1,29%
Atrial fibrillation	4135	1,23%
Ischemic heart disease/ heart attack	3885	1,16%
Heart failure	3782	1,13%
Musculoskeletal	30,630	9,14%
Low back pain/Sciatica	9939	2,96%
Fractures	8966	2,67%
Musculoskeletal pain	4800	1,43%
Gastrointestinal	22,972	6,85%
Abdominal pain	9479	2,83%
Gastritis/ Dyspepsia	7208	2,15%
Neurological	12,305	3,67%
Dementia	3742	1,12%
Stroke	3410	1,02%
E.N.T.	12,112	3,61%
Vertigo	9250	2,76%
Psychiatric	10,060	3,00%
Anxiety disorder/ Panic attack	6183	1,84%
Respiratory tract	6282	1,87%
COPD	3482	1,04%
Genito-urinary tract	5622	1,68%
Skin/mucosal diseases	6266	1,87%
Symptoms	11,458	3,42%
Fever	5969	1,78%
Weakness/fatigue	2550	0,76%
Dehydration	1838	0,55%
Oedema	1101	0,33%
Medical procedures	20,020	5,97%
Placement of urinary catheters	8952	2,67%
Placement of naso-gastric tubes	3080	0,92%
Bed-sore treatment	2650	0,79%
Radiology	9405	2,81%
X-rays	5609	1,67%
Ultrasound	3021	0,90%
Triplex	775	0,23%
Follow up examination	7118	2,12%
No specific diagnosis recorded	5255	1,57%
Other^a^	24,303	7.25
Missing data	19,467	5,81%
Total	335,212	100,00%

^a^include eye disorders, endocrine/metabolic diseases, acid-base disturbances, cancer, burns, intoxication, bites, minor trauma, allergies, venous disorders, hematological diseases, adverse drug reactions, death certificates, and routine visits that each presented with frequency < 1%

The changes in working diagnoses among the age groups with the higher number of calls during the study period are shown in Table 4. In the age group 76-90 years a positive correlation was shown for ear-nose-throat disorders and a negative for infections, cardiovascular and gastrointestinal disorders; no correlation was seen for all other disease categories. In the age group > 90 years a positive correlation was observed for ear-nose-throat and respiratory disorders and a negative for cardiovascular, gastrointestinal and skin disorders; no correlation was seen for all other conditions.

**Table 4:** Correlation between the percentage of diseases and the percentage of calls in each age group during the study period

Diseases	Age group 75-90			Age group > 90		
	r	*p*-value	interpretation	r	*p*-value	interpretation
Infections	−0,88	< 0,001	negative	−0,56	0,075	none
Cardiovascular	−0,83	0,002	negative	−0,94	< 0,001	negative
Musculoskeletal	−0,48	0,131	none	−0,36	0,278	none
Gastrointestinal	−0,79	0,004	negative	−0,61	0,047	negative
Neurological	0,22	0,520	none	0,44	0,179	none
E.N.T.	0,9	< 0,001	positive	0,8	0,003	positive
Psychiatric	−0,008	0,981	none	−0,39	0,237	none
Respiratory tract	0,58	0,064	none	0,63	0,037	positive
Genito-urinary tract	0,31	0,348	none	0,41	0,210	none
Skin/mucosal diseases	−0,52	0,099	none	−0,67	0,020	negative

Another approach employed by the SOS network during this period was radiology at home. Improvements in health technology enabled the performance of more than 9400 visits for X-rays (59%) and ultrasounds (including triplex studies); 99% of these visits were performed from 2009 onwards. An increasing demand for such visits was observed, starting at 352 calls in 2009 and reaching 2230 in 2015.

Data regarding patient satisfaction were available for 23,306 follow up phone calls. The majority of them were performed during the years 2013-2015 (19,211, 82.4%). In general, patient satisfaction rate was high (97.7%). The most common etiology for lack of satisfaction was doctor’s behavior (discontent 1%, rudeness 0.9% or the patient’s perception that the doctor did not devote the expected time for his/her condition 0.1%). Incorrect diagnosis was an uncommon reason for patient’s discontent (0.2%). There were not major discrepancies in satisfaction rate within age categories; the higher satisfaction rate was observed in patients 76-90 years (98.1%) and the lower in patients 30-45 years (97.3%).

## Discussion

A continuous shift in the age of the population utilizing the SOS doctors’ network was observed. An increasing number of patients requesting home visits in the age group 60-90 was observed, particularly for the second half of this age group. On the contrary, the number of patients in the other age groups and particularly those ≥91 and < 16 years old declined in time. In this study, approximately 70% of patients were over 60 years old. In a previous analysis, the respective proportion was approximately 51% [12]. These changes may partially reflect the change in demographics of the aging population in Greece, or a higher need for follow up visits. However, this could not account for the unexpected decrease in home visits for the most frail, those older than 90 years old, as home health care is proportional to the number of chronic conditions and the functional impairments [15]. On the other hand, it may reflect a change in the preferences or priorities among different patient populations or close relatives providing care to older or disabled patients. For example, the current financial crisis in Greece affected mainly younger ages (increase of unemployment and immigration, lower wages), while older individuals were primarily affected through a decrease in pensions.

The preferred medical specialty was also studied in association with patients’ age. Patients examined by internists and general practitioners were younger than those examined by all other specialties. An explanation for this could be the higher prevalence of chronic diseases with advancing age. This has been shown in a study in the USA [16]. In that study, the mean age of patients at cardiology practices was higher than that in internal and family medicine. In addition these patients had also more co-morbidity and worse functional status. The system of care was also related to patients’ age in that study [16]. Another explanation could be the decline in the percentage of home visits for infections with advancing age seen in our study (from approximately 65% in the age group 16-30 years to < 20% in those aged over 90, data not shown).

Significant changes in the gender and timing of the received calls were not observed between the current and a previous analysis. Females requested the majority of the calls in both periods [12]. Most of the calls were performed during office hours, with 2 evident peaks, identical in the two analyses [12]. On the other hand, we observed changes in the distribution of the etiology of the calls. Although infections remained the primary cause, their proportion was reduced to 29% (compared to 33.6% in the period 2001-2005). More importantly, since 2009 an almost 50% reduction in the calls for infections has been observed. An increase in the proportion of calls for cardiovascular diseases, musculoskeletal complaints, gastrointestinal diseases, neurological, respiratory and skin diseases was seen. In contrast calls for ear-nose-throat disorders, psychiatric, and genitourinary diseases were reduced.

The SOS doctors network in Greece covered mainly for acute conditions (infections, uncontrolled pain of multiple origin, vertigo etc), or acute exacerbations of chronic diseases. Follow up and routine evaluations had subtle contribution in home visits. This can be attributed to the cost (the service is not covered by the public primary health care provider in Greece), habits of the population or the physician, or the difficulty in tracking and following up patients with chronic diseases at home (physicians’ schedule, coordination, trip planning issues, chronic conditions require files for follow up). On the other hand, it may reflect the perception of patients regarding the diseases that can be managed at home or may not require further investigation or hospitalization. It may also reflect conditions for which patients are willing to pay for (out-of-pocket service for acute, often debilitating or quality of life modifying conditions in comparison to stable chronic diseases). Finally, this may also reflect different approaches in healthcare management, as for example, post-discharge requests for continuing intravenous therapy at home were largely absent.

A peak in the number of calls was observed in 2009, and thereafter the number of calls dropped annually to reach a plateau in the years 2013-2015. As the majority of calls were due to infections of the respiratory tract, it could be suggested that the increase in the number of calls in 2009 could be attributed to the influenza pandemic observed in this year. Although the number of calls due to respiratory tract infections was not very different during that period compared to other years, non-infectious complications of influenza may have equally contributed to the increase in home visits [17].

Someone could argue that the financial crisis in Greece could be the main reason behind this fall during the initial period (2010-2013) of the financial crisis. However, other reasons may also apply. For example, a home visit may reflect a need for disabled individuals or convenience for wealthier, younger patients. Therefore, the chief complaint or the underlying disease or patient condition may not justify the physician’s visit. The sudden decrease in calls for respiratory tract infections (especially upper ones) denotes that convenience and house comfort could be among the main drive for the increase in the number of calls up to 2009. In this regard, the financial crisis might have contributed to fewer calls for mild to moderate or self limited (from the patient/community view) diseases.

Hospital admission was recommended in approximately 9% of visits. Thus, home visits prevented a significant number of emergency department visits. Other reasons for declining hospital services included the patient’s or their first degree relative’s refusal to go to the hospital and advanced directives and preferences on goals of care. A previous patient’s will to receive treatment at home instead of a hospital [12], is well documented in the medical literature [18]. Additionally, caregivers expressed occasionally their concerns regarding the quality of care their old relative would receive in the hospital.

In most countries of the developed world, several companies or organizations (including hospitals and insurers) offer house-call visits mainly by general practitioners and family physicians; other services can include nurse practitioners (an increasingly used service in the USA), nurses, social workers, psychologists, or a multidisciplinary approach providing care with 2 or more of the aforementioned professionals [19, 20]. In contrast to the worldwide practice, the SOS network in Greece offers house-call visits by specialists. Although internists perform the great majority of visits, most of the remaining specialties are also available and their share in the care of patients, especially of the elderly, is increasing. This innovative approach may have advantages and disadvantages. The advantage is the high quality of services provided by specialists in their field. The disadvantage could be the higher cost for the visit, especially for countries like Greece in which the insurers do not reimburse for the visits.

Home visits are expected to increase over the following years for reasons pertaining to physicians’, community’s/state’s and patients’ interests. Patients specific causes include older age, frailty, disabilities that affect not only their capability to walk but also to perform their daily activities, and terminal illnesses (cancer, heart failure, neurologic diseases). In this regard, immobility or short walking distance ability, cognitive impairment, psychological/emotional disorders, need for assessment of home care (environment, care givers), or severe current status of the patients (unconsciousness, severe debilitation from dehydration/paresis/hemodynamic instability) can justify a home-visit. Furthermore, house calls can strengthen the relationship between the physician and the patient.

Difficulties in providing healthcare services at home for acute and chronic conditions include the need to carry the necessary supplies (drugs for parenteral administration or non-oral routes especially for homebound individuals, sets for intravenous administration of fluids and drugs, wound care dressings and cleansers), cooperation with nurses and non-medical staff (physical therapist, dietician, speech therapist, social workers), and possibly the cost for the patients (the service in Greece is out-of-pocket, with partial coverage in case of selected patients with private insurance; in our study only 1.5% of patients had their visit paid by a private insurance).

Recently published studies showed that young physicians dismiss home visits [21]. Besides financial incentives, key factors that could enhance the physician’s willingness to participate in a home call include override of barriers like schedules and travel time, personal safety and liability, and further development of applications (diagnostic devices/tests, communication with doctors of other specialties) through technological advances that could increase the degree of certainty in working diagnosis [1, 2]. Home visits enhance the ability of the physician to understand the patients’ needs and environment. The structure and organization of SOS doctors, the exploitation of available resources as well as the competitive compensation provide a means to overcome several of the obstacles that may prevent a physician to perform home visits. Help by professional networks like the SOS doctors or similar agencies around the world could provide effective guidance and help to primary care physicians. The expertise provided by specialized physicians is a unique feature of the Greek SOS network and a significant opportunity for the expansion of home visits.

In addition, home healthcare can reduce the overall cost of healthcare; it may also improve the quality of life of the patients and their families by reducing unnecessary hospitalizations. Using historical data, the Alliance for Home Health Quality and Innovation forecasted potential savings of $10.3 billion over 10 years (2014-2023) in the USA by reducing regional hospital readmissions through the use of home health as the first line to handle post-acute care episodes [22]. Furthermore, when patients can stay home, hospital beds and personnel can focus on the most acute cases. Depending on healthcare economics, other countries could probably achieve relevant savings. Studies showed that patients prefer to receive healthcare services in the comfort and privacy of their own homes for the treatment of an acute or chronic condition [18]. Finally, lowering the cost by preventing frail, prone to iatrogenic complications patients from reaching more expensive treatment settings, e.g. hospitals, would be the strongest motive for governments to develop home-based, physician assisted health care programs [10, 23, 24].

It is difficult to assess or compare the effectiveness of house call visits in individual studies as it depends on several factors, including the frailty of the patients, the targeted intervention, the outcome measure, the study design and the composition of the treating team. Thus, it was not surprising that published reviews, meta-analyses and individual studies on e.g. the effectiveness of preventive home visits to the elderly, came up with conflicting results [5, 25–28]. The usefulness of such programs should not be judged only by their effectiveness on measurable outcomes but also on their ability to meet special social needs. In fact, such services may be considered mainly as a social process than a treatment program [29]. On the other hand, hospital-level care can be effectively delivered to carefully selected, older patients with acute illnesses at their homes [23]. In addition, it was the preferred modality of care among older patients, with high patient and family member satisfaction rate [30].

The patient satisfaction rate after home visits by both physicians and non-physicians has been documented by several studies [3, 30, 31]. In a previous study, home visits satisfaction rate was high and increased by early physician arrival and younger age of the patient. Other commonly encountered factors, such as gender, family income, employment or marital status do not seem to affect patients’ satisfaction rate [32]. In the present study the satisfaction rate was very high (97.7%) without major discrepancies according to patient’s age. The prompt arrival of the physician and the relatively low cost of the service should have increased the satisfaction rate. Whether this could be also attributed to the utilization of specialists instead of general practitioners should be evaluated in comparative studies in the future.

The study findings are limited by the lack of data regarding the patient population that could ask for this out-of-pocket medical service. Although potentially all inhabitants in the greater metropolitan areas of Athens and Thessaloniki could use the service, the number of patients willing to receive it due to financial and other reasons cannot be accurately estimated. Other factors like population aging, emergence of companies providing similar services and changes in the private economy (including private insurance holders which were < 3% throughout the study period) in Greece during the crisis might have influenced the findings. On the other hand, the organization and function of SOS Doctors did not change substantially throughout the study period besides changes in the number of participating doctors and the increased demand for radiology services.

## Conclusion

This analysis provides insights to the requests for home visits by specialists in Greece. Despite the ongoing financial crisis the demand for home visits did not decrease substantially. However, it might have affected the characteristics of patients as well as the etiology for the service request, as a shift towards older age, but not the oldest old (> 90 years), and acute conditions was observed. The study confirms that home visits remain an option for health services in the modern health care systems.
